# Supplementary material for: The gut microbiota-bile acid axis links the positive association between chronic insomnia and cardiometabolic diseases
Source: Nat Commun. 2022 May 30;13:3002. doi: 10.1038/s41467-022-30712-x (PMC9151781; doi:10.1038/s41467-022-30712-x)
Supplement: Supplementary file 1 — Supplementary Information [file 41467_2022_30712_MOESM1_ESM.pdf]

Supplementary Information

"The gut microbiota-bile acid axis links the positive association between chronic insomnia and cardiometabolic diseases"

This supplementary file contains the following:

Supplementary Methods:

Method S1 Fecal microbial DNA extraction and 16S rRNA gene sequencing in the Guangzhou Nutrition and Health Study

Method S2 16S rRNA gene sequencing bioinformatics in the Guangzhou Nutrition and Health Study

Method S3 Targeted fecal bile acid profiling in the Guangzhou Nutrition and Health Study

Supplementary Tables:

Supplementary Table 1 Gut microbiota features of chronic insomnia with three different models

Supplementary Table 2 The stratification analysis of the chronic insomnia-gut microbiota association by age or sex

Supplementary Table 3 The association of chronic insomnia symptom score with *Ruminococcaceae UCG-002* and *Ruminococcaceae UCG-003* in the Guangdong Gut Microbiome Project

Supplementary Table 4 Sensitivity analysis for the association of chronic insomnia with bile acids by adding dietary cholesterol intake and fiber intake as additional covariates

Supplementary Table 5 The association of chronic insomnia with short-chain fatty acids, aromatic amino acids and their derivatives

Supplementary Table 6 The stratification analysis of the gut microbiota-CMD association by age or sex

Supplementary Table 7 Sensitivity analysis of the mediation effect of bile acids in the association of chronic insomnia-related gut microbiota with CMD risk.
Supplementary Table 8 The association of tea consumption with chronic insomnia

Supplementary Figures:

Supplementary Fig. 1 The association of chronic insomnia with Shannon index, Chao 1 index, ACE index and Simpson index among four groups. n = 1,809. **a**. Shannon index for chronic insomnia. **b**, Chao 1 index for chronic insomnia. **c**. ACE index for chronic insomnia. **d**, Simpson index for chronic insomnia. Multivariable linear regression was used to estimate the difference in Shannon index, Chao 1 index, ACE index and Simpson index among four groups with three different statistical models (Methods). Box plots indicate median and interquartile range (IQR). The upper and lower whiskers indicate 1.5 times the IQR from above the upper quartile and below the lower quartile. Value with symbol is significantly different (model 1: $^{*}p < 0.05$, $^{**}p < 0.01$, $^{***}p < 0.001$; model 2: $^{+}p < 0.05$, $^{++}p < 0.01$, $^{+++}p < 0.001$; model 3: $^{\#}p < 0.05$, $^{\#\#}p < 0.01$, $^{\#\#\#}p < 0.001$). All statistical tests were two-sided. Source data are provided as a Source Data file.

Supplementary Fig. 2 The association of chronic insomnia with Shannon index, Chao 1 index, ACE index and Simpson index in the discovery cohort comparing Chronic insomnia group and Long-term healthy group. n = 1,809. **a**. Shannon index for chronic insomnia. **b**, Chao 1 index for chronic insomnia. **c**. ACE index for chronic insomnia. **d**, Simpson index for chronic insomnia. Multivariable linear regression was used to estimate the difference in Shannon index, Chao 1 index, ACE index and Simpson index comparing chronic insomnia group and Long-term healthy group using three different statistical models (Methods). Box plots indicate median and interquartile range (IQR). The upper and lower whiskers indicate 1.5 times the IQR from above the upper quartile and below the lower quartile. Value with symbol is significantly different (model 1: $^{*}p < 0.05$, $^{**}p < 0.01$, $^{***}p < 0.001$; model 2: $^{+}p <$

0.05, $^{++}p < 0.01$, $^{+++}p < 0.001$; model 3: $^{\#}p < 0.05$, $^{\#\#}p < 0.01$, $^{\#\#\#}p < 0.001$). All statistical tests were two-sided. Source data are provided as a Source Data file.

Supplementary Fig. 3 The association of chronic insomnia with Shannon index, Chao 1 index, ACE index and Simpson index in the validation cohort comparing Chronic insomnia group and Non-chronic insomnia group. n = 6,122. **a**. Shannon index for chronic insomnia. **b**, Chao 1 index for chronic insomnia. **c**. ACE index for chronic insomnia. **d**, Simpson index for chronic insomnia. Multivariable linear regression was used to estimate the difference in Shannon index, Chao 1 index, ACE index and Simpson index comparing Chronic insomnia group and Non-chronic insomnia group, adjusted for age, sex, BMI, smoking status, alcohol status, education, dietary intake of vegetables, fruits, and red and processed meat. Box plots indicate median and interquartile range (IQR). The upper and lower whiskers indicate 1.5 times the IQR from above the upper quartile and below the lower quartile. Value with asterisk is significantly different ($^{*}p < 0.05$, $^{**}p < 0.01$, $^{***}p < 0.001$). All statistical tests were two-sided. Source data are provided as a Source Data file.

Supplementary Fig. 4 Sensitivity analysis for the association between chronic insomnia and gut microbial features in the Guangdong Gut Microbiome Project. For the Guangdong Gut Microbiome Project (GGMP), we did not include income in the statistical models due to large amount of missing values (income data were available among 3774 out of 6122 participants). A sensitivity analysis using multivariable linear regression (β coefficient) with further adjustment for income was used to examine the robustness of the associations between chronic insomnia and gut microbial features. Values presented are beta coefficients (95% confidence intervals). Value with asterisk is significantly different ($^{*}p < 0.05$, $^{**}p < 0.01$, $^{***}p < 0.001$). Error bars are beta coefficient with 95% confidence intervals. The Benjamini-Hochberg method was used to control false discovery rate (FDR) due to multiple testing. All statistical tests were two-sided. Source data are provided as a Source Data file.

Supplementary Fig. 5 The potential bile acid features of chronic insomnia. Orthogonal partial least squares discrimination analysis (OPLS-DA) was used to identify potential bile acids associated with chronic insomnia comparing Chronic insomnia group with Long-term healthy group. The x axis shows the variable important in projection (VIP) values and the y axis indicates the partial correlation coefficient values. Points are coloured based on the significance of the obtained associations (green indicates positive associations with VIP >1 and $|p\ (\mathrm{corr})| > 0.3$, pink indicates negative associations with VIP >1 and $|p\ (\mathrm{corr})| > 0.3$). All statistical tests were two-sided. Source data are provided as a Source Data file.

Supplementary Fig. 6 Sensitivity analysis for the association between chronic insomnia-related microbial features and cardiometabolic diseases in the Guangdong Gut Microbiome Project. In the Guangdong Gut Microbiome Project (GGMP), income data were available among 3774 out of 6122 participants. A sensitivity analysis using Multivariable logistic regression (odds ratio) with further adjustment for income was used to estimate the association of the chronic insomnia inverse-related microbial features *Ruminococcaceae UCG-002* and *Ruminococcaceae UCG-003* with different cardiometabolic diseases (CMD) in the discovery and validation cohorts, respectively (Methods). The effect estimates from the discovery and validation cohorts were pooled using random effects meta-analysis for each of the above analyses. Values presented are odds ratio (95% confidence intervals) with corresponding p-values. Value with asterisk is significantly different (*$p < 0.05$, ** $p < 0.01$, ***$p < 0.001$). The Benjamini-Hochberg method was used to control FDR due to multiple testing. All statistical tests were two-sided. Source data are provided as a Source Data file.

Supplementary Fig. 7 Prospective association of dietary factors with the identified microbial feature *Ruminococcaceae UCG-003* linking chronic insomnia and cardiometabolic diseases. Prospective association of dietary factors with the

identified microbial feature *Ruminococcaceae UCG-003* linking chronic insomnia and cardiometabolic diseases (CMD) in the GMHS. Multivariable linear regression was used to determine the prospective association of dietary factors with *Ruminococcaceae UCG-003*, adjusting for age, sex, BMI, smoking status, alcohol status, physical activity, education, income, dietary intake of vegetables/fruits/red and processed meat/fish/dairy products/coffee/tea) (mutual adjustment for each other) and total energy intake. The analyses were conducted among the GNHS participants without chronic insomnia or CMD at baseline. Values presented are beta coefficients (95% confidence intervals) with corresponding *p*-values. Value with asterisk is significantly different (*$p < 0.05$, ** $p < 0.01$, ***$p < 0.001$). The Benjamini-Hochberg method was used to control FDR due to multiple testing. All statistical tests were two-sided. Source data are provided as a Source Data file.

Supplemental Methods

Method S1 Fecal microbial DNA extraction and 16S rRNA gene sequencing in the Guangzhou Nutrition and Health Study

Fecal microbial DNA was extracted from each sample using the QIAamp® DNA Stool Mini Kit (Qiagen, Hilden, Germany) following the manufacturer's instruction. DNA concentration and purity were monitored on 1% agarose gels. According to the concentration, DNA was diluted to 1 ng/μL using sterile water. The 16S rRNA gene amplification procedure was divided into two PCR steps, in the first PCR reaction, the V3-V4 hypervariable region of the 16S rRNA gene was amplified from genomic DNA using primers 341F(CCTACGGGNGGCWGCAG) and 805R(GACTACHVGGGTAT CTAATCC). Amplification was performed in 96-well microtiter plates with a reaction mixture consisting of 1X KAPA HiFi Hot start Ready Mix, 0.1μM primer 341 F, 0.1 μM primer 805 R, and 12.5 ng template DNA, giving a total volume of 50 μL per sample. Reactions were run in a T100 PCR thermocycle (BIO-RAD) according to the following cycling program: 3 min of denaturation at 94 °C, followed by 18 cycles of 30 s at 94 °C (denaturing), 30 s at 55 °C (annealing), and 30 s at 72 °C (elongation), with a final extension at 72 °C for 5 min. Subsequently, the amplified products were checked by 2% agarose gel electrophoresis and ethidium bromide staining. Amplicons were quantified using the Qubit quantification system (Thermo Scientific, Wilmington, DE, US) following the manufacturers' instructions. Sequencing primers and adaptors were added to the amplicon products in the second PCR step as follows 2 μL of the diluted amplicons were mixed with a reaction solution consisting of 1×KAPA HiFi Hotstart ReadyMix, 0.5μM fusion forward and 0.5μM fusion reverse primer, 30 ng Meta-gDNA(total volume 50 μL). The PCR was run according to the cycling program above except with cycling number of 12. The amplification products were purified with Agencourt AMPure XP Beads (Beckman Coulter Genomics, MA, USA) according to the manufacturer's instructions and quantified as described above. Equimolar amounts of the amplification products were pooled together in a single tube. The concentration of the pooled libraries was determined by the Qubit quantification system. Amplicon sequencing was performed on the Illuimina MiSeq

System (Illumina Inc., CA, USA). The MiSeq Reagent Kits v2 (Illumina Inc.) was used. Automated cluster generation and 2 × 250 bp paired-end sequencing with dual-index reads were performed.

Method S2 16S rRNA gene sequencing bioinformatics in the Guangzhou Nutrition and Health Study

Fastq-files were demultiplexed by the MiSeq Controller Software (Bcl2fastq version 2.20, Illumina Inc.). The sequences were trimmed for amplification primers, diversity spacers, and sequencing adapters, merge-paired and quality filtered by Quantitative Insights into Microbial Ecology (QIIME) software version 2-2020.2. DADA2 was used for amplicon sequence variants (ASVs) clustering equaling 100%[1]. A representative sequence was picked for each ASV and the Sliva reference database version 138 was used to annotate taxonomic information. α-diversity (Observed species, Shannon index, Chao1 index) and β-diversity (Bray-Curtis distance) measures were calculated based on the genus-level counts. The genus-level absolute abundance table was extracted from the pipeline and converted to relative abundances by normalizing for analyzing the composition of gut microbiota by QIIME2.

Principal coordinate analysis (PCoA) based on genus-level Bray-Curtis distance and permutational multivariate analysis of variance (PERMANOVA) were performed to compare the dissimilarity of global microbiota composition. MaAsLin is a multivariate statistical framework that identifies associations between continuous and discrete clinical metadata and microbial community abundances. Microbiome-based biomarker discovery was performed with MaAsLin using the online galaxy server (https://huttenhower.sph.harvard.edu/galaxy/). Briefly, the percentage of each genus was arcsin-square-root-transformed. For each correlation between metadata (as predictors) and transformed genus abundances (as response variables), we adjusted potential confounders using the three different statistical models (q value (false discovery rate adjusted p value) ≤ 0.25 was considered statistically significant). Model 1 was adjusted for age, sex, BMI, smoking status, alcohol status, physical

activity, education, income and total energy intake at baseline. Model 2 was further adjusted for hypertension, hyperlipidaemia, metabolic syndrome (MetS), type 2 diabetes (T2D), coronary heart disease (CHD), stroke and medication for T2D. Model 3 was further adjusted for vegetables, fruits, red and processed meat, fish, dairy products, coffee and tea. To further reduce the computational load, the genera that were present in < 10% of the population were excluded. The Benjamini-Hochberg method was used to adjust p values for multiple hypotheses.

Method S3 Targeted fecal bile acid profiling in the Guangzhou Nutrition and Health Study

Targeted bile acid profiling of fecal samples (n=954) was performed by Metabo-Profile (Shanghai, China). The order of all test samples is randomly selected before the preparation. 10 mg lyophilized feces were homogenized with 25μL water and extracted with 185μL cold ACN-Methanol (8/2, v/v). After centrifugation, 30 μL supernatant was used to derivatization with 20 μL freshly prepared derivative reagents on a Biomek 4000 workstation (Biomek 4000, Beckman Coulter, Inc., Brea, California, USA), followed by mixing with internal standards. Subsequently, the derivatized samples and serial dilutions of derivatized stock standards were randomly analyzed and quantitated by an ultra-performance liquid chromatography coupled to tandem mass spectrometry (UPLC-MS/MS) system (ACQUITY UPLC-Xevo TQ-S, Waters Corp., Milford, MA, USA). 41 of bile acid standard substances were obtained from Sigma-Aldrich (St. Louis, MO, USA), Steraloids Inc. (Newport, RI, USA) and TRC Chemicals (Toronto, ON, Canada). Three types of quality control samples i.e., test mixtures, internal standards, and pooled biological samples are routinely used in metabolomics platform. The derivatized pooled quality control samples were injected every 14 test samples. Raw data generated by UPLC-MS/MS were processed using the QuanMET software (v2.0, Metabo-Profile, Shanghai, China) to perform peak integration, calibration, and quantification for each bile acid.

Supplementary Tables

Supplementary Table 1 Gut microbiota features of chronic insomnia with three different models*

Genus	Coefficient	Correlation direction	*q* value	Phylum	Family
Model 1					
Ruminococcaceae UCG-002	-0.077	Negative	0.112	Firmicutes	Ruminococcaceae
Ruminococcaceae UCG-003	-0.050	Negative	0.112	Firmicutes	Ruminococcaceae
Model 2					
Ruminococcaceae UCG-002	-0.077	Negative	0.140	Firmicutes	Ruminococcaceae
Ruminococcaceae UCG-003	-0.052	Negative	0.130	Firmicutes	Ruminococcaceae
Model 3					
Ruminococcaceae UCG-002	-0.073	Negative	0.191	Firmicutes	Ruminococcaceae
Ruminococcaceae UCG-003	-0.049	Negative	0.223	Firmicutes	Ruminococcaceae

*The gut microbiota biomarkers were determined by MaAsLin adjusted by three different models. Model 1 was adjusted for age, sex, BMI, smoking status, alcohol status, physical activity, education, income and total energy intake at baseline. Model 2 was further adjusted for hypertension, hyperlipidaemia, metabolic syndrome (MetS), type 2 diabetes (T2D), coronary heart disease (CHD), stroke and medication for T2D. Model 3 was further adjusted for vegetables, fruits, red and processed meat, fish, dairy products, coffee and tea. q value < 0.25 was considered statistically significant.

Supplementary Table 2 The stratification analysis of the chronic insomnia-gut microbiota association by age or sex*

Cohorts	Beta coefficient	95%CI	*p* value	$p_{interaction}$ value
Age stratification				
Ruminococcaceae UCG-002				0.447
High age group				
Discovery cohort (n = 856)	-0.25	[-0.44, -0.05]	0.012	
Validation cohort (n = 3024)	-0.10	[-0.17, -0.03]	0.008	
Meta-analysis (n = 3880)	-0.15	[-0.28, -0.01]	0.032	
Low age group				
Discovery cohort (n = 863)	-0.14	[-0.31, 0.04]	0.120	
Validation cohort (n = 3098)	-0.07	[-0.14, -0.01]	0.047	
Meta-analysis (n = 3961)	-0.08	[-0.15, -0.02]	0.015	
Ruminococcaceae UCG-003				0.562
High age group				
Discovery cohort (n = 856)	-0.19	[-0.35, -0.04]	0.017	
Validation cohort (n = 3024)	-0.10	[-0.17, -0.04]	0.008	
Meta-analysis (n = 3880)	-0.12	[-0.19, -0.05]	0.001	
Low age group				
Discovery cohort (n = 863)	-0.12	[-0.26, 0.02]	0.092	
Validation cohort (n = 3098)	-0.06	[-0.14, 0.01]	0.078	
Meta-analysis (n = 3961)	-0.08	[-0.14, -0.01]	0.019	
Sex stratification				
Ruminococcaceae UCG-002				0.127
men				
Discovery cohort (n = 571)	-0.21	[-0.50, 0.08]	0.155	
Validation cohort (n = 2723)	-0.07	[-0.15, 0.01]	0.063	

Meta-analysis (n = 3294)	-0.08	[-0.16, -0.01]	0.031	
women				
Discovery cohort (n = 1148)	-0.19	[-0.33, -0.04]	0.013	
Validation cohort (n = 3399)	-0.11	[-0.17, -0.04]	0.003	
Meta-analysis (n = 4547)	-0.12	[-0.18, -0.06]	<0.001	
Ruminococcaceae UCG-003				0.904
men				
Discovery cohort (n = 571)	-0.23	[-0.46, -0.01]	0.040	
Validation cohort (n = 2723)	-0.04	[-0.12, 0.03]	0.289	
Meta-analysis (n = 3294)	-0.11	[-0.29, 0.07]	0.240	
women				
Discovery cohort (n = 1148)	-0.14	[-0.26, -0.02]	0.024	
Validation cohort (n = 3399)	-0.11	[-0.18, -0.04]	0.001	
Meta-analysis (n = 4547)	-0.12	[-0.18, -0.06]	<0.001	

* Multivariable linear regression (Beta coefficient) was used to estimate the association of chronic insomnia with *Ruminococcaceae UCG-002* and *Ruminococcaceae UCG-003*. Median of age was used as the cutoff in the age stratification. All statistical tests were two-sided.

Supplementary Table 3 The association of chronic insomnia symptom score with *Ruminococcaceae UCG-002* and *Ruminococcaceae UCG-003* in the Guangdong Gut Microbiome Project*

Species	Beta coefficient	95%CI	*p* value
Ruminococcaceae UCG-002	-0.04	[-0.06, -0.02]	<0.001
Ruminococcaceae UCG-003	-0.04	[-0.07, -0.01]	0.002

*Multivariable linear regression (Beta coefficient) was used to estimate the association of chronic insomnia symptom score with the gut microbial biomarkers of chronic insomnia, adjusted for the same covariates. All statistical tests were two-sided.

Supplementary Table 4 Sensitivity analysis for the association of chronic insomnia with bile acids by adding dietary cholesterol intake and fiber intake as additional covariates*

Bile acids	Beta coefficient	95%CI	*p* value
Muro cholic acid	0.21	[0.04, 0.38]	0.049
Nor cholic acid	0.21	[0.04, 0.37]	0.042
Isolithocholic acid	-0.26	[-0.43, -0.09]	0.030
Lithocholic acid	-0.21	[-0.38, -0.04]	0.035
Ursodeoxycholic acid	-0.22	[-0.39, -0.05]	0.049

*Multivariable linear regression (Beta coefficient) was used to estimate the association of chronic insomnia with bile acids, adjusted for dietary cholesterol intake and fiber intake as additional covariates in the above model 3. The Benjamini-Hochberg method was used to control false discovery rate (FDR) for multiple testing. All statistical tests were two-sided.

Supplementary Table 5 The association of chronic insomnia with short-chain fatty acids, aromatic amino acids and their derivatives*

Microbial metabolites	Beta coefficient	95%CI	*p* value
Short chain fatty acids			
Acetic acid	0.01	[-0.03, 0.04]	0.899
Propionic acid	0.01	[-0.04, 0.05]	0.978
Butyric acid	-0.01	[-0.05, 0.04]	0.909
Isobutyric acid	-0.03	[-0.09, 0.02]	0.079
Valeric acid	-0.04	[-0.13, 0.04]	0.649
Isovaleric acid	-0.03	[-0.10, 0.04]	0.595
Isocaproic acid	-0.06	[-0.18, 0.06]	0.787
Caproic acid	-0.12	[-0.25, 0.01]	0.547
Aromatic amino acids and their derivatives			
L-tyrosine	0.01	[-0.04, 0.05]	0.799
4-hydroxyphenylpyruvic acid	-0.01	[-0.07, 0.05]	0.816
4-hydroxyphenylacetic acid	0.05	[-0.04, 0.15]	0.549
4-hydroxyphenyllactic acid	-0.01	[-0.10, 0.10]	0.965
4-hydroxybenzoic acid	0.07	[-0.02, 0.16]	0.534
Hippuric acid	0.14	[-0.04, 0.32]	0.502
Ortho-hydroxyphenylacetic acid	-0.04	[-0.14, 0.05]	0.662
L-phenylalanine	0.01	[-0.04, 0.05]	0.803
Phenylpyruvic acid	-0.05	[-0.14, 0.04]	0.761
Phenyllactic acid	-0.08	[-0.16, 0.01]	0.781
Phenylacetic acid	-0.05	[-0.14, 0.04]	0.541
2-phenylpropionate	-0.16	[-0.36, 0.03]	0.781
L-tryptophan	0.02	[-0.03, 0.06]	0.762
3-indolepropionic acid	0.10	[-0.27, 0.07]	0.634

Indoleacetic acid	-0.03	[-0.12, 0.07]	0.735

*Multivariable linear regression (Beta coefficient) was used to estimate the association of chronic insomnia with short-chain fatty acids, aromatic amino acids and their derivatives, adjusted for same covariates as above model 3. The Benjamini-Hochberg method was used to control false discovery rate (FDR) for multiple testing. All statistical tests were two-sided.

Supplementary Table 6 The stratification analysis of the gut microbiota-CMD association by age or sex*

Cohorts	Odds ratio (OR)	95%CI	*p* value	$p_{interaction}$ value
Age stratification				
Ruminococcaceae UCG-002-Dyslipidaemia				0.270
High age group				
Discovery cohort (n = 856)	0.88	[0.77, 1.02]	0.083	
Validation cohort (n = 3024)	0.90	[0.84, 0.97]	0.005	
Meta-analysis (n = 3880)	0.90	[0.84, 0.96]	0.001	
Low age group				
Discovery cohort (n = 863)	0.85	[0.73, 0.99]	0.036	
Validation cohort (n = 3098)	0.99	[0.92, 1.06]	0.717	
Meta-analysis (n = 3961)	0.93	[0.73, 1.07]	0.314	
Ruminococcaceae UCG-002-T2D				0.489
High age group				
Discovery cohort (n = 856)	0.82	[0.68, 0.99]	0.039	
Validation cohort (n = 3024)	0.84	[0.76, 0.94]	0.003	
Meta-analysis (n = 3880)	0.84	[0.76, 0.92]	<0.001	
Low age group				
Discovery cohort (n = 863)	0.70	[0.52, 0.91]	0.011	
Validation cohort (n = 3098)	1.02	[0.87, 1.20]	0.792	
Meta-analysis (n = 3961)	0.86	[0.59, 1.25]	0.426	
Ruminococcaceae UCG-002-MetS				0.713
High age group				
Discovery cohort (n = 856)	0.70	[0.58, 0.84]	<0.001	
Validation cohort (n = 3024)	0.90	[0.83, 0.98]	0.017	
Meta-analysis (n = 3880)	0.81	[0.63, 1.03]	0.089	

Low age group				
Discovery cohort (n = 863)	0.84	[0.69, 1.01]	0.072	
Validation cohort (n = 3098)	0.85	[0.77, 0.95]	0.003	
Meta-analysis (n = 3961)	0.85	[0.78, 0.93]	<0.001	
Ruminococcaceae UCG-003-Dyslipidaemia				0.511
High age group				
Discovery cohort (n = 856)	0.91	[0.79, 1.04]	0.173	
Validation cohort (n = 3024)	0.89	[0.83, 0.95]	0.001	
Meta-analysis (n = 3880)	0.89	[0.84, 0.95]	<0.001	
Low age group				
Discovery cohort (n = 863)	0.84	[0.72, 0.98]	0.028	
Validation cohort (n = 3098)	0.88	[0.82, 0.95]	<0.001	
Meta-analysis (n = 3961)	0.87	[0.82, 0.93]	<0.001	
Ruminococcaceae UCG-003-T2D				0.834
High age group				
Discovery cohort (n = 856)	0.92	[0.77, 1.09]	0.367	
Validation cohort (n = 3024)	0.85	[0.76, 0.96]	0.007	
Meta-analysis (n = 3880)	0.87	[0.79, 0.96]	0.006	
Low age group				
Discovery cohort (n = 863)	0.71	[0.52, 0.94]	0.026	
Validation cohort (n = 3098)	0.95	[0.80, 1.12]	0.555	
Meta-analysis (n = 3961)	0.85	[0.64, 1.12]	0.239	
Ruminococcaceae UCG-003-MetS				0.631
High age group				
Discovery cohort (n = 856)	0.70	[0.57, 0.84]	<0.001	
Validation cohort (n = 3024)	0.88	[0.81, 0.96]	0.003	
Meta-analysis (n = 3880)	0.80	[0.64, 0.99]	0.047	

Low age group				
Discovery cohort (n = 863)	0.86	[0.70, 1.05]	0.148	
Validation cohort (n = 3098)	0.81	[0.73, 0.90]	<0.001	
Meta-analysis (n = 3961)	0.82	[0.75, 0.90]	<0.001	
Sex stratification				
Ruminococcaceae UCG-002-Dyslipidaemia				0.003
men				
Discovery cohort (n = 571)	0.82	[0.68, 0.98]	0.033	
Validation cohort (n = 2723)	0.88	[0.81, 0.95]	<0.001	
Meta-analysis (n = 3294)	0.87	[0.81, 0.93]	<0.001	
women				
Discovery cohort (n = 1148)	0.80	[0.68, 1.02]	0.109	
Validation cohort (n = 3399)	0.99	[0.93, 1.07]	0.892	
Meta-analysis (n = 4547)	0.96	[0.88, 1.05]	0.394	
Ruminococcaceae UCG-002-T2D				0.158
men				
Discovery cohort (n = 571)	0.90	[0.71, 1.13]	0.377	
Validation cohort (n = 2723)	0.93	[0.82, 1.06]	0.287	
Meta-analysis (n = 3294)	0.92	[0.82, 1.04]	0.173	
women				
Discovery cohort (n = 1148)	0.70	[0.57, 0.86]	<0.001	
Validation cohort (n = 3399)	0.85	[0.75, 0.97]	0.014	
Meta-analysis (n = 4547)	0.79	[0.66, 0.95]	0.011	
Ruminococcaceae UCG-002-MetS				0.082
men				
Discovery cohort (n = 571)	0.75	[0.60, 0.93]	0.009	

Validation cohort (n = 2723)	0.84	[0.76, 0.92]	<0.001	
Meta-analysis (n = 3294)	0.82	[0.76, 0.90]	<0.001	
women				
Discovery cohort (n = 1148)	0.77	[0.65, 0.90]	0.002	
Validation cohort (n = 3399)	0.77	[0.65, 0.90]	0.080	
Meta-analysis (n = 4547)	0.85	[0.71, 1.02]	0.075	
Ruminococcaceae UCG-003-Dyslipidaemia				0.120
men				
Discovery cohort (n = 571)	0.75	[0.61, 0.91]	0.004	
Validation cohort (n = 2723)	0.85	[0.79, 0.92]	<0.001	
Meta-analysis (n = 3294)	0.82	[0.74, 0.92]	<0.001	
women				
Discovery cohort (n = 1148)	0.96	[0.85, 1.08]	0.464	
Validation cohort (n = 3399)	0.92	[0.84, 1.01]	0.016	
Meta-analysis (n = 4547)	0.93	[0.87, 0.99]	0.014	
Ruminococcaceae UCG-003-T2D				0.222
men				
Discovery cohort (n = 571)	0.96	[0.75, 1.20]	0.719	
Validation cohort (n = 2723)	0.92	[0.80, 1.05]	0.202	
Meta-analysis (n = 3294)	0.93	[0.82, 1.04]	0.199	
women				
Discovery cohort (n = 1148)	0.80	[0.65, 0.98]	0.036	
Validation cohort (n = 3399)	0.85	[0.75, 0.97]	0.019	
Meta-analysis (n = 4547)	0.84	[0.75, 0.94]	0.002	
Ruminococcaceae UCG-003-MetS				0.367
men				
Discovery cohort (n = 571)	0.81	[0.64, 1.00]	0.064	

Validation cohort (n = 2723)	0.80	[0.73, 0.88]	<0.001
Meta-analysis (n = 3294)	0.80	[0.74, 0.88]	<0.001
women			
Discovery cohort (n = 1148)	0.76	[0.63, 0.89]	<0.001
Validation cohort (n = 3399)	0.90	[0.82, 0.99]	0.030
Meta-analysis (n = 4547)	0.84	[0.71, 0.99]	0.041

*Multivariable logistic regression (Odds ratio) was used to estimate the association of *Ruminococcaceae UCG-002* and *Ruminococcaceae UCG-003* with cardiometabolic disease (CMD) risk. Median of age was used as the cutoff in the age stratification. All statistical tests were two-sided. T2D: type 2 diabetes; MetS: metabolic syndrome.

Supplementary Table 7 Sensitivity analysis of the mediation effect of bile acids in the association of chronic insomnia-related gut microbiota with CMD risk*

Sensitivity results		Outcomes
Ruminococcaceae UCG-002		
		MetS
For MCA:	Rho at which ACME = 0	0.100
For NorCA:	Rho at which ACME = 0	0.130
		T2D
For IsoLCA:	Rho at which ACME = 0	-0.120
Ruminococcaceae UCG-003		
		MetS
For MCA:	Rho at which ACME = 0	0.130
For NorCA:	Rho at which ACME = 0	0.145
		T2D
For IsoLCA:	Rho at which ACME = 0	-0.145

*The sensitivity analysis of mediation was performed using the R package “medsens” with default parameters. The Rho from the R package “medsens” is interpreted as a Pearson correlation. For example, a Rho of 0.10 is often regarded as a weak association, which implies that it would take some set of unmeasured confounders of the mediator and outcome to induce a relatively weak correlation between the mediator and the outcome for the observed mediated effect to equal zero. MCA: Muro cholic acid; NorCA: nor cholic acid; IsoLCA: isolithocholic acid; CMD: cardiometabolic disease; MetS: metabolic syndrome; T2D: type 2 diabetes; ACME: average causal mediated effect.

Supplementary Table 8 The association of tea consumption with chronic insomnia*

Cohorts	Odds ratio (OR)	95%CI	*p* value
Discovery cohort	0.85	[0.60, 1.19]	0.334
Validation cohort	0.64	[0.49, 0.83]	<0.001
Meta-analysis	0.72	[0.55, 0.95]	0.020

*Multivariable logistic regression (Odds ratio) was used to estimate the association of tea consumption with chronic insomnia, adjusted for the potential covariates. The Benjamini-Hochberg method was used to control false discovery rate (FDR) for multiple testing. All statistical tests were two-sided.

Supplementary Figures

Supplementary Fig. 1 The association of chronic insomnia with Shannon index, Chao 1 index, ACE index and Simpson index among four groups.

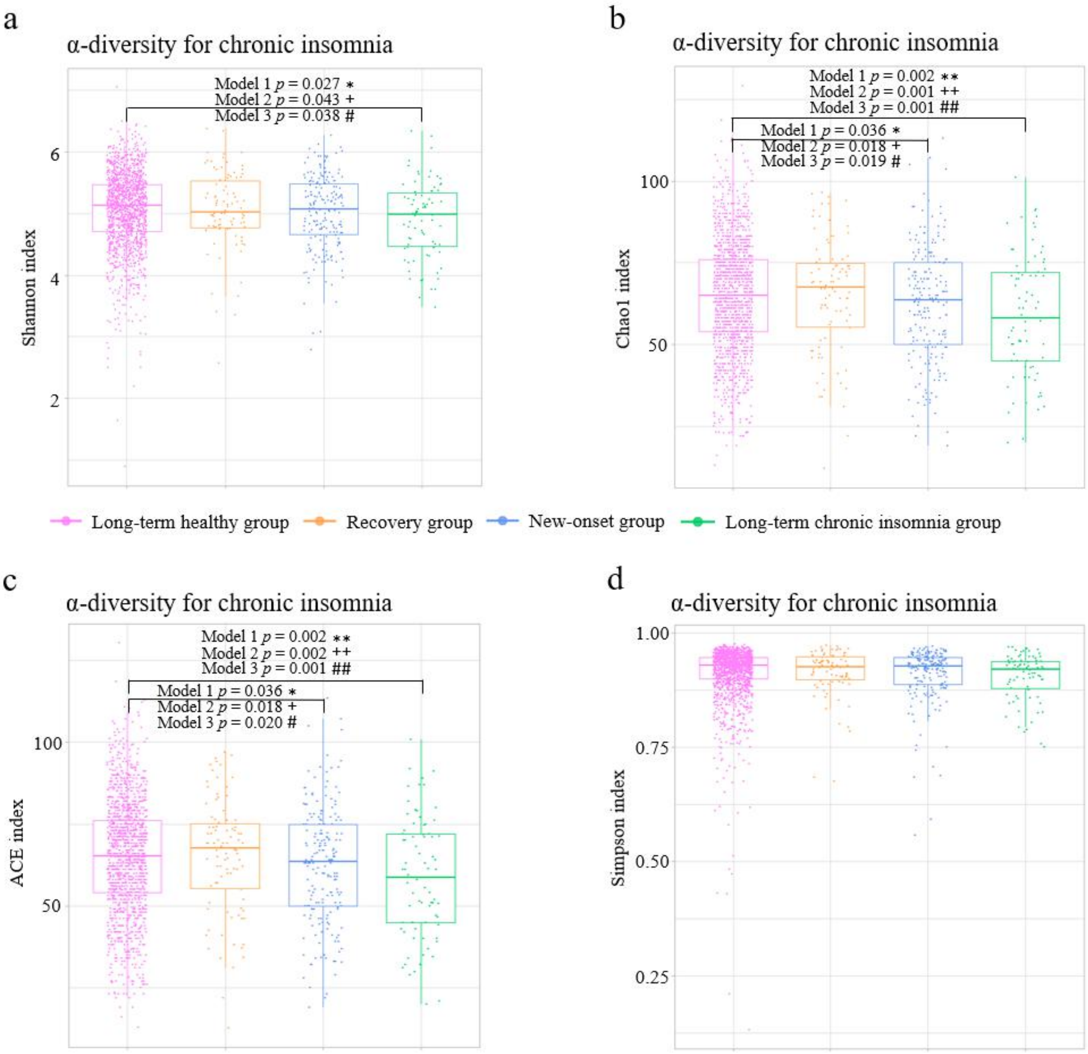

Supplementary Fig. 2 The association of chronic insomnia with Shannon index, Chao 1 index, ACE index and Simpson index in the discovery cohort comparing Chronic insomnia group and Long-term healthy group.

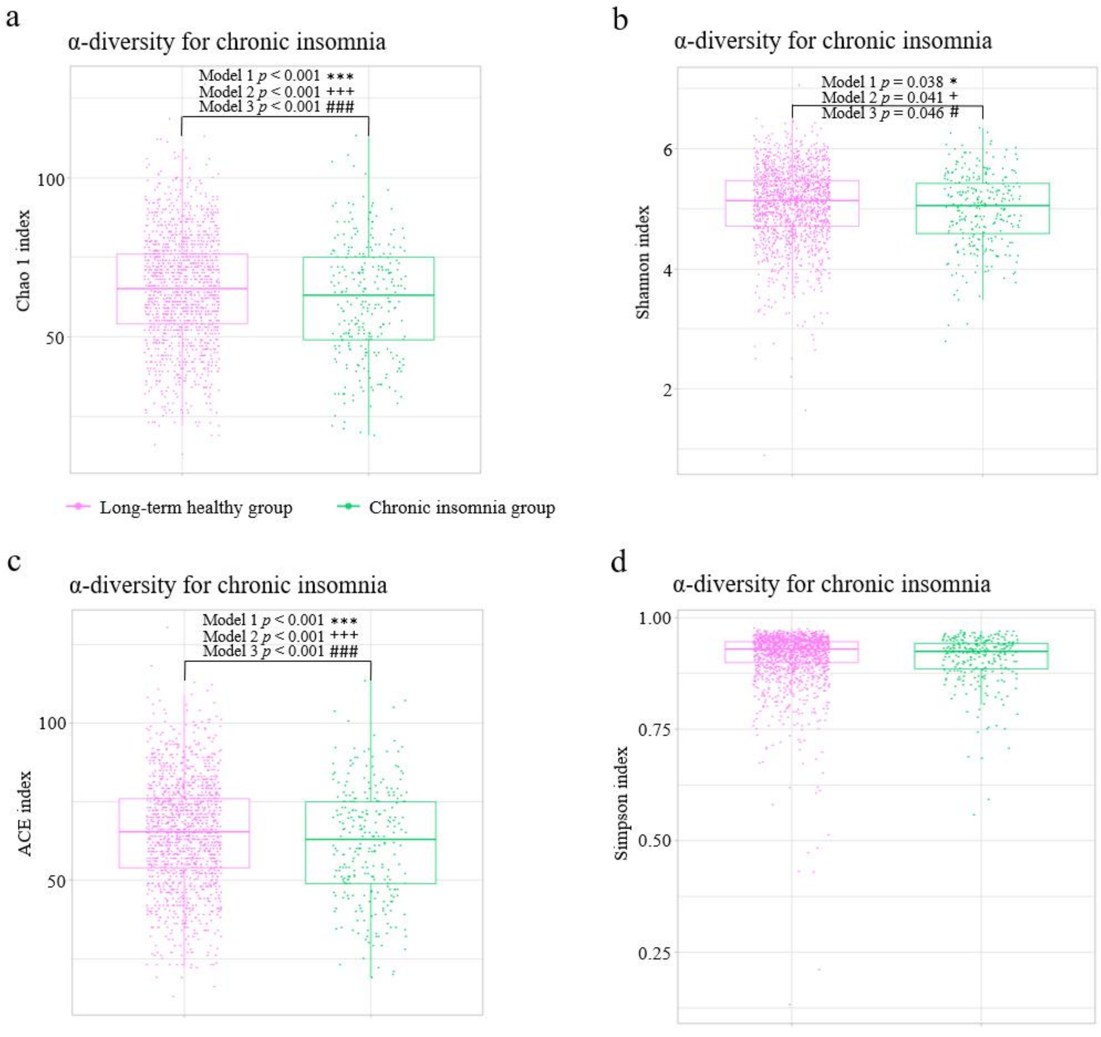

Supplementary Fig. 3 The association of chronic insomnia with Shannon index, Chao 1 index, ACE index and Simpson index in the validation cohort comparing Chronic insomnia group and Non-chronic insomnia group.

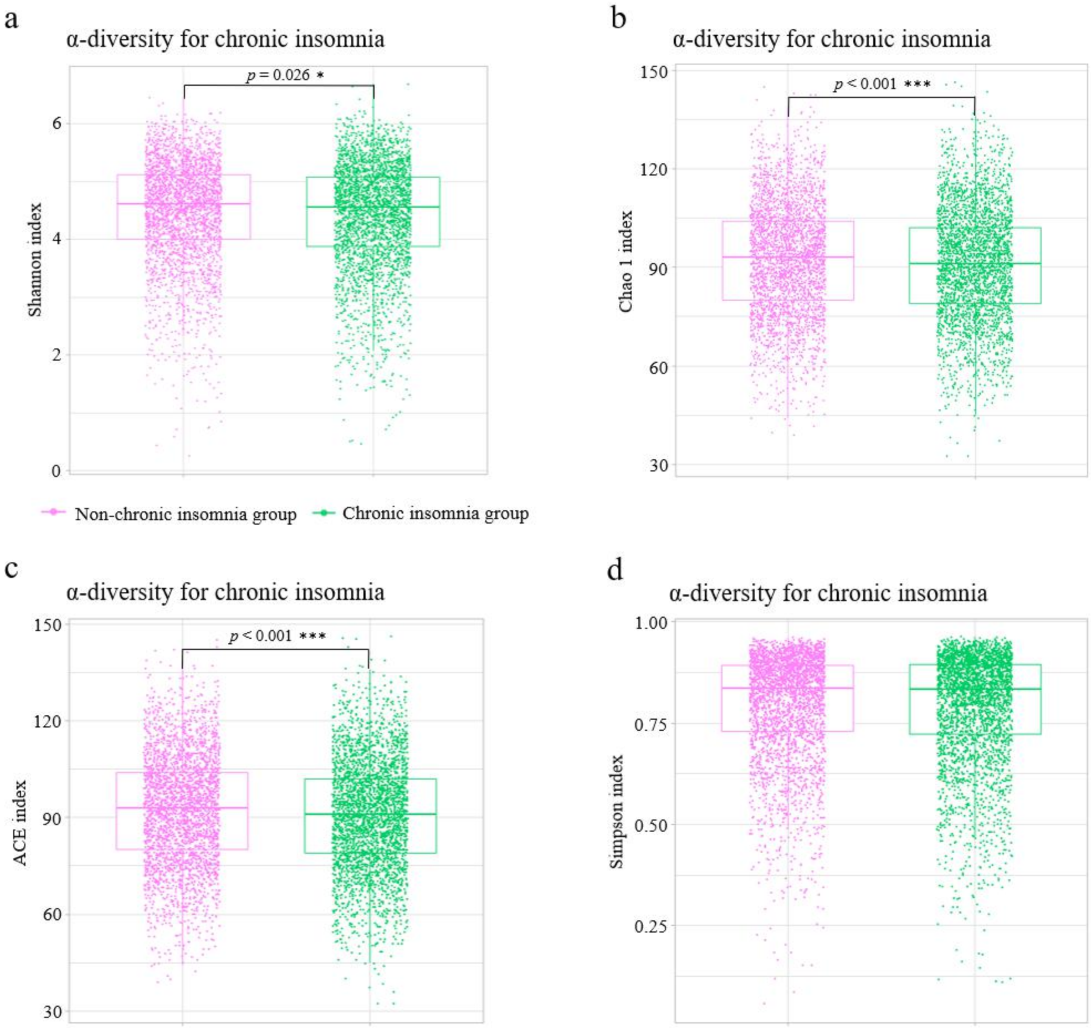

Supplementary Fig. 4 Sensitivity analysis for the association between chronic insomnia and gut microbial features in the Guangdong Gut Microbiome Project.

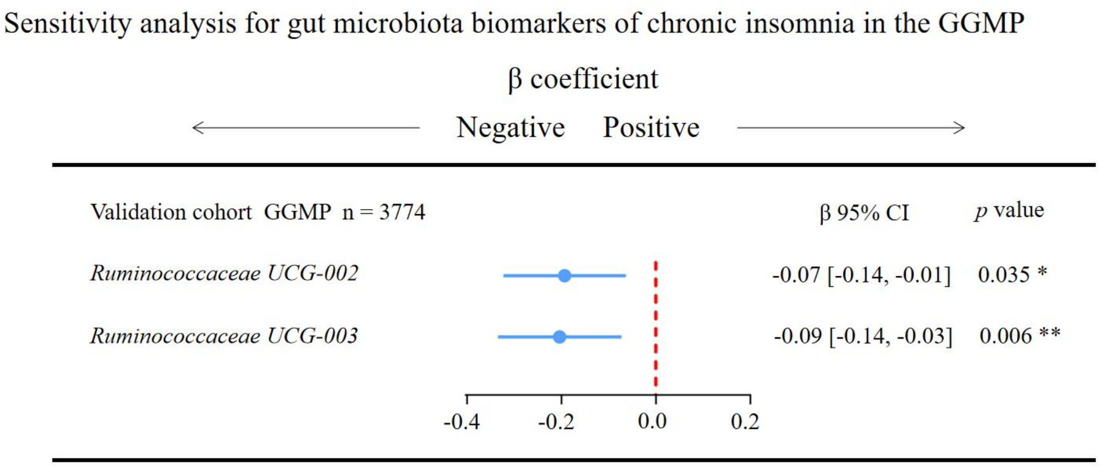

Supplementary Fig. 5 The potential bile acid features of chronic insomnia.

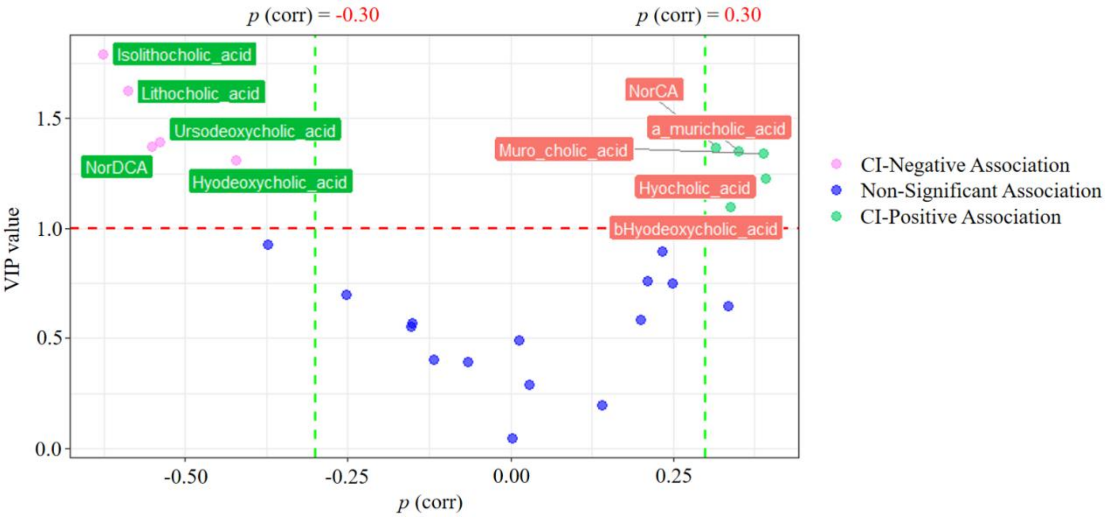

Supplementary Fig. 6 Sensitivity analysis for the association between chronic insomnia-related microbial features and cardiometabolic diseases in the Guangdong Gut Microbiome Project.

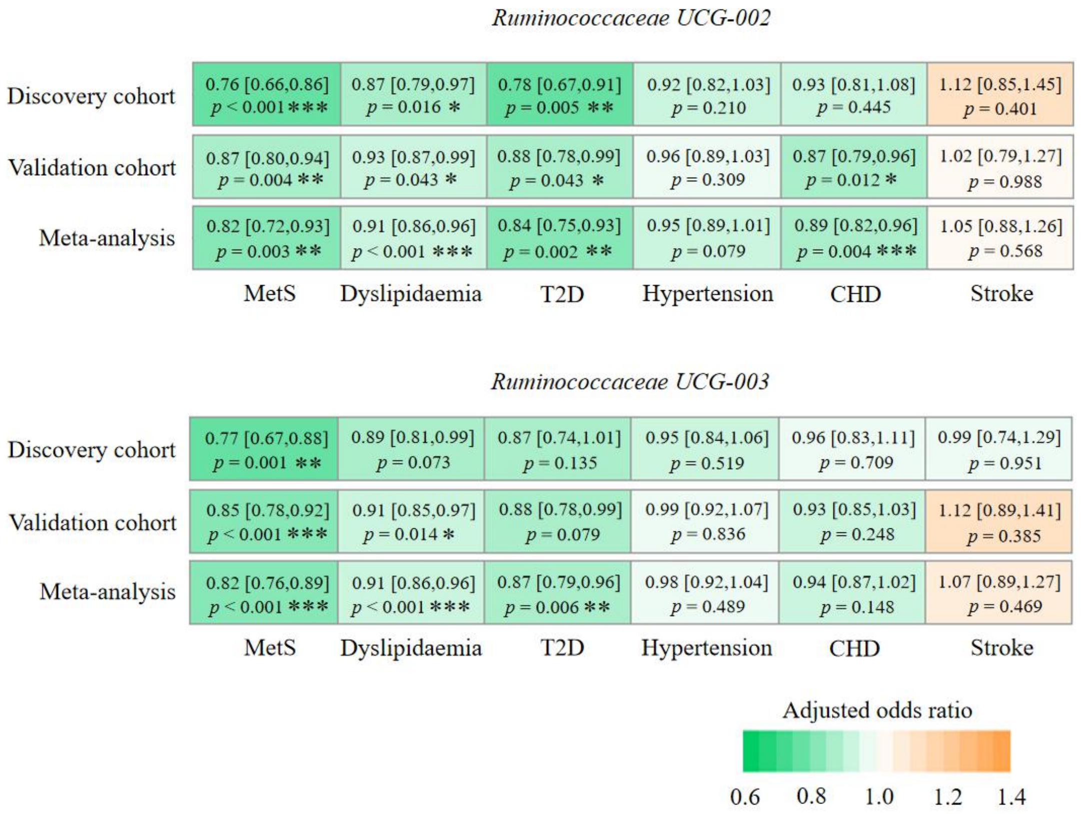

Supplementary Fig. 7 Prospective association of dietary factors with the identified microbial feature *Ruminococcaceae UCG-003* linking chronic insomnia and cardiometabolic diseases.

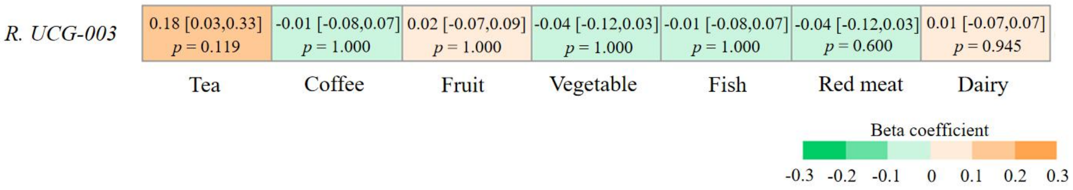

Reference

1. Bolyen E*, et al.* Reproducible, interactive, scalable and extensible microbiome data science using QIIME 2. *Nat Biotechnol* **37**, 852-857 (2019).
