# Supplementary material for: The gut microbiota-bile acid axis links the positive association between chronic insomnia and cardiometabolic diseases
Source: Nat Commun. 2022 May 30;13:3002. doi: 10.1038/s41467-022-30712-x (PMC9151781; doi:10.1038/s41467-022-30712-x)
Supplement: Supplementary file 3 — Reporting Summary [file 41467_2022_30712_MOESM3_ESM.pdf]

nature portfolio

Corresponding author(s): Ju-Sheng Zheng

Last updated by author(s): Apr 28, 2022

Reporting Summary

Nature Portfolio wishes to improve the reproducibility of the work that we publish. This form provides structure for consistency and transparency in reporting. For further information on Nature Portfolio policies, see our Editorial Policies and the Editorial Policy Checklist.

Statistics

For all statistical analyses, confirm that the following items are present in the figure legend, table legend, main text, or Methods section.

n/a	Confirmed	
☐	☒	The exact sample size (*n*) for each experimental group/condition, given as a discrete number and unit of measurement
☐	☒	A statement on whether measurements were taken from distinct samples or whether the same sample was measured repeatedly
☐	☒	The statistical test(s) used AND whether they are one- or two-sided<br>*Only common tests should be described solely by name; describe more complex techniques in the Methods section.*
☐	☒	A description of all covariates tested
☐	☒	A description of any assumptions or corrections, such as tests of normality and adjustment for multiple comparisons
☐	☒	A full description of the statistical parameters including central tendency (e.g. means) or other basic estimates (e.g. regression coefficient) AND variation (e.g. standard deviation) or associated estimates of uncertainty (e.g. confidence intervals)
☐	☒	For null hypothesis testing, the test statistic (e.g. *F*, *t*, *r*) with confidence intervals, effect sizes, degrees of freedom and *P* value noted<br>*Give P values as exact values whenever suitable.*
☒	☐	For Bayesian analysis, information on the choice of priors and Markov chain Monte Carlo settings
☐	☒	For hierarchical and complex designs, identification of the appropriate level for tests and full reporting of outcomes
☐	☒	Estimates of effect sizes (e.g. Cohen's *d*, Pearson's *r*), indicating how they were calculated

Our web collection on statistics for biologists contains articles on many of the points above.

Software and code

Policy information about availability of computer code

Data collection	QuanMET software version 2.0 has been used for targeted fecal metabolomics data collection. MiSeq Controller Software version (Bcl2fastq version 2.20) has been used for 16S rRna gene sequencing data collection.
Data analysis	MiSeq Controller Software (Bcl2fastq version 2.20); Quantitative Insights into Microbial Ecology (QIIME) software version 2-2020.2; Multivariate Analysis by Linear Models (MaAsLin) using the online galaxy server (https://huttenhower.sph.harvard.edu/galaxy/); QuanMET software version 2.0; Cytoscape software version 3.7.2; ChemDraw version 10.0; R version 3.6.3, R packages (ggplot2 version 3.3.5, ggpubr version 0.4.0, pheatmap version 1.0.12 and mediation version 4.5.0) and Sliva reference database version 138.

For manuscripts utilizing custom algorithms or software that are central to the research but not yet described in published literature, software must be made available to editors and reviewers. We strongly encourage code deposition in a community repository (e.g. GitHub). See the Nature Portfolio guidelines for submitting code & software for further information.

Data

Policy information about availability of data

All manuscripts must include a data availability statement. This statement should provide the following information, where applicable:

- Accession codes, unique identifiers, or web links for publicly available datasets
- A description of any restrictions on data availability
- For clinical datasets or third party data, please ensure that the statement adheres to our policy

16S rRNA gene sequencing data of the Guangzhou Nutrition and Health Study (GNHS) are available in the Genome Sequence Archive (GSA) (https://ngdc.cncb.ac.cn/gsa/) at accession number CRA006769. 16S rRNA gene sequencing data of the Guangdong Gut Microbiome Project (GGMP) are available from the European Nucleotide Archive (https://www.ebi.ac.uk/ena/) at accession number PRJEB18535. The Sliva reference database version 138 was used to annotate taxonomic

information. The metadata of the GGMP are available in a previous publication (https://pubmed.ncbi.nlm.nih.gov/30250144/). The data associated with this study are presented in the paper, supplementary information and Source Data file. Source data are provided with this paper.
Codes used for this study are available at: https://github.com/nutrition-westlake/Chronic-insomnia-Gut-microbiota-bile-acid-axis-and-Cardiometabolic-diseases-Project/blob/main/Code%20available.

Field-specific reporting

Please select the one below that is the best fit for your research. If you are not sure, read the appropriate sections before making your selection.

☒ Life sciences ☐ Behavioural & social sciences ☐ Ecological, evolutionary & environmental sciences

For a reference copy of the document with all sections, see nature.com/documents/nr-reporting-summary-flat.pdf

Life sciences study design

All studies must disclose on these points even when the disclosure is negative.

Sample size	No sample size calculation was performed because the present study is observational, not interventional.
Data exclusions	The Guangzhou Nutrition and Health Study (GNHS) is a community-based prospective cohort conducted between 2008 and 2013 including 4048 participants of Han Chinese ethnicity. Fecal samples of the participants were collected at the second follow-up at the study site up to Apr 30, 2019 (median follow-up of 6.2 years from entry into the cohort). We excluded participants who were (1) without measurement of gut microbiota data (n=2125); (2) without valid questionnaire information on chronic insomnia (n=4); (3) with self-reported baseline cancers, chronic renal dysfunction, or cirrhosis (n=71); (4) with missing covariates (age, sex, BMI, education, income, smoking status, alcohol status, total energy intake and physical activity) (n=24); (5) with extreme levels of dietary total energy intake (men: < 800 kcal or > 4000 kcal; women: < 500 kcal or > 3500 kcal) (n=15). Finally, 1809 participants were included in the present analysis.<br>The Guangdong Gut Microbiome Project (GGMP) is a large community-based cross-sectional cohort conducted between 2015 and 2016 including 7009 participants with high quality gut microbiome data. We excluded participants (1) without chronic insomnia information (n=633); (2) with missing covariates (age, sex, BMI, education, smoking status, alcohol status) (n=254). Finally, we included 6122 participants (52.8 ± 14.7 y, 55.2% of women) from the GGMP in our analysis as an independent validation cohort.
Replication	Gut microbial features of chronic insomnia and their relationships with cardiometabolic disease traits were replicated in the independent cohort (GGMP). The association of habitual dietary intake with gut microbial features of chronic insomnia was also validated in the GGMP.
Randomization	Not applicable for this observational study.
Blinding	Not applicable for this observational study.

Reporting for specific materials, systems and methods

We require information from authors about some types of materials, experimental systems and methods used in many studies. Here, indicate whether each material, system or method listed is relevant to your study. If you are not sure if a list item applies to your research, read the appropriate section before selecting a response.

Materials & experimental systems

n/a	Involved in the study	
☒	☐	Antibodies
☒	☐	Eukaryotic cell lines
☒	☐	Palaeontology and archaeology
☒	☐	Animals and other organisms
☐	☒	Human research participants
☒	☐	Clinical data
☒	☐	Dual use research of concern

Methods

n/a	Involved in the study	
☒	☐	ChIP-seq
☒	☐	Flow cytometry
☒	☐	MRI-based neuroimaging

Human research participants

Policy information about studies involving human research participants

Population characteristics	We used two human cohorts in the present study: the Guangzhou Nutrition and Health Study (GNHS, n = 1,809)(Table 1) and the Guangdong Gut Microbiome Project (GGMP, n = 6,122) (Table 2), who provided detailed information on chronic insomnia status, cardiometabolic disease and related risk factors .
Recruitment	The GNHS is a community-based prospective cohort. The participants, 40-75 years old and living in southern China, Guangzhou City, were recruited into the GNHS between 2008 and 2013. The GGMP is a large community-based cross-sectional cohort conducted between 2015 and 2016. The GGMP participants were from 14 randomly selected districts or counties in Guangdong province, China. All the participants from the two cohorts provided written informed consent. This study is based on the observational study design, residual confounders could not be avoided.
Ethics oversight	The study protocol of GNHS was approved by the Ethics Committee of the School of Public Health at Sun Yat-sen University and Ethics Committee of Westlake University. The study protocol of GGMP was approved by the Ethical Review Committee of Chinese Center for Disease Control and Prevention.

Note that full information on the approval of the study protocol must also be provided in the manuscript.
